# MODIMA, a Method for Multivariate Omnibus Distance Mediation Analysis, Allows for Integration of Multivariate Exposure–Mediator–Response Relationships

**DOI:** 10.3390/genes10070524

**Published:** 2019-07-11

**Authors:** Bashir Hamidi, Kristin Wallace, Alexander V. Alekseyenko

**Affiliations:** 1 Program for Human Microbiome Research, Medical University of South Carolina, 135 Cannon Street MSC 200, Charleston, SC 29425, USA; 2 Biomedical Informatics Center, Medical University of South Carolina, 135 Cannon Street MSC 200, Charleston, SC 29425, USA; 3 Department of Public Health Science, Medical University of South Carolina, 135 Cannon Street MSC 200, Charleston, SC 29425, USA; 4 Department of Oral Health Sciences, Medical University of South Carolina, 135 Cannon Street MSC 200, Charleston, SC 29425, USA; 5 Department of Healthcare Leadership and Management, Medical University of South Carolina, 135 Cannon Street MSC 200, Charleston, SC 29425, USA

**Keywords:** multivariate analysis, multivariate causal mediation, distance correlation, direct effect, indirect effect, causal inference

## Abstract

Many important exposure–response relationships, such as diet and weight, can be influenced by intermediates, such as the gut microbiome. Understanding the role of these intermediates, the mediators, is important in refining cause–effect theories and discovering additional medical interventions (e.g., probiotics, prebiotics). Mediation analysis has been at the heart of behavioral health research, rapidly gaining popularity with the biomedical sciences in the last decade. A specific analytic challenge is being able to incorporate an entire ’omics assay as a mediator. To address this challenge, we propose a hypothesis testing framework for multivariate omnibus distance mediation analysis (MODIMA). We use the power of energy statistics, such as partial distance correlation, to allow for analysis of multivariate exposure–mediator–response triples. Our simulation results demonstrate the favorable statistical properties of our approach relative to the available alternatives. Finally, we demonstrate the application of the proposed methods in two previously published microbiome datasets. Our framework adds a new tool to the toolbox of approaches to the integration of ‘omics big data.

## 1. Introduction

Natural biological phenomena are often explained using statistical methods by means of isolating the individual contexts of the phenomenon itself by establishing associations. For example, obesity, among other factors, maybe related to changes in nutrition or stress. Although these explanations fail to present a full account of the original observed phenomenon or capture the entirety of such complex dynamics, they aid in our understanding of the cause–effect relationships, especially when a plausible causal directionality can be established (e.g., increase in calorie consumption is plausibly causal to weight gain, and not the other way around). The next level of complexity is afforded by incorporating additional mechanisms arising from one or more other intermediate factors. In order to properly understand the mechanisms involved, we must understand the extent to which the exposure of interest (calorie intake) directly affects an outcome (weight gain) and the extent to which the exposure indirectly affects the outcome through intermediate factors (e.g., gut microbiome) [1,2,3,4]. Mediation analysis is at the heart of many human behavior studies and is quickly gaining traction in the biomedical research arena. With the explosion growth of the ‘omics, we see the development of new analysis and tools that provide access to the integration of new knowledge and their applications as mediators of treatment–effect relationships.

Microbiome research has advanced significantly in the last decade with the rise of computational power, next-generation sequencing, and data analytics [5,6]. Naturally arising have been translational investigations assessing the interplay of human–host microbial communities with various health and diseases states [7,8,9], yet a notable challenge remains of understanding the extent to which and mechanisms by which such interactions take place. Three notable interactions comprise this dynamic relationship: first, the association between the environment and the host; second, the association between the microbiome and host health or disease; and third, the association between the environment and the microbiome. Because of this complexity, most available observational and experimental study designs are unable to properly assess direct causal roles of the microbiome, and, in many cases, alternative interpretations are plausible. We have seen a growing volume of evidence linking microbiome and human disease such as that of obesity, inflammatory bowel disease, and colorectal cancer [10,11]. Similarly, we have seen the relationship between environmental factors and the microbiome [12]. Now, we believe it is important to assess how outside environmental factors or host genetic characteristics affect the microbiome and, together with changes of microbiome composition, influence human health and disease. Accordingly, there is an urgent need for statistical methods that establish and isolate the mediation role of microbes in these complex dynamics.

Formal approaches to the assessment of mediation effects are primarily based on the work by Baron and Kenny [13] using the product of coefficients. The single mediator model (SMM) describes the relationship between exposure (X), response (Y), and a mediator (M), each of which are univariate random variables. SMM posits that the relationship between those can be described in terms of linear regressions, that capture the effect of the exposure on the response:

(1) $$\begin{array}{c} Y=\;i_{1}+\gamma X+\varepsilon_{1},\; \end{array}$$

the effect of the exposure on the mediator:

(2) $$\begin{array}{c} M=\;i_{3}+\alpha X+\varepsilon_{3}, \end{array}$$

and the effect of both on the response:

(3) $$\begin{array}{c} Y=\;i_{2}+\gamma^{\prime }X+\beta M+\varepsilon_{2}. \end{array}$$

The downside of the conceptual simplicity of the linear regression-based framework is the lack of a convenient test that could allow for the evaluation of the hypotheses about the presence of mediation without the need to estimate the regression coefficients. To this end, Boca et al. [14] have provided a mediation testing framework that casts the regression equations in terms of correlations and partial correlations. They further propose a multiple testing framework for the evaluation of the hypotheses related to the presence of multiple mediators. However, the omnibus mediation hypothesis still lacks an acceptable simple solution. Furthermore, no current approach allows for multivariate exposures and responses.

Microbiome analytics must take into account the multivariate nature of such data, and thus often use distance-based approaches. In these cases, power and type I error characteristics are often directly related to the chosen distance metric. Some proposed methods such as the work of Zhao et al. [15] utilize multiple distance and dissimilarity metrics in a regression-based association testing framework. As another example, Tang et al. [6] assessed true association by using multiple distances simultaneously and by allowing the flexible adjustment of confounders through computing residuals by regression of the covariates on confounders. As we have seen in published reviews, limitations exist to proposed methods upon the application to ‘omics data [16]. One such case is microbiome data, which are high-dimensional, under-sampled, compositional, and over-dispersed; nonetheless, we would like to be able to explain their role as a mediator just like a univariate mediator would. Within this article, we present a framework for multivariate distance mediation analysis that is suitable to such data.

In this article, we present a framework for testing multivariate distance mediation to allow for multivariate exposures, responses, and mediators. We build our test on the mediation approach published by Boca et al. [14] and extend it to high-dimensional data via distance-based methodologies. We present simulation results on the robustness and sensitivity of the proposed methods and further make comparisons with other proposed approaches, such as permutation-based testing by Boca et al. [14] and sample-wise distance matrices by Zhang et al. [17]. Lastly, we analyze two real datasets to demonstrate the power of the proposed methods and their application to high-dimensional microbiome data.

## 2. Materials and Methods

### 2.1. Availability and Implementation

Supplementary materials include reference implementation of the methods, simulation studies, and application examples and are freely available at https://github.com/alekseyenko/MODIMA.

### 2.2. Testing for Mediation

The testing framework developed by Boca et al. [14] expresses the relationships captured in the SMM linear regressions in terms of Pearson correlations. Thus, for a significant effect of the exposure on the response to exist, the correlation between the two has to be non-zero, $\rho \left(X,\;Y\right)>0$. Furthermore, if the relationship is in fact mediated by M, both the correlation between exposure and the mediator and the conditional correlation of the mediator and the response, given the exposure, should be non-zero [13], $\rho \left(X,\;M\right)>0$ and $\rho \left(r_{M|X},\;r_{Y|X}\right)>0$, respectively. Here, $r_{M|X}\;and\;r_{Y|X}$ denote the residuals of the conditional correlation on regression of *X* on *M* and *X* on *Y*, respectively. These observations give rise to the following test statistic:

(4) $$S\left(X,M,Y\right)=\rho \left(X,\;M\right)\;\rho \left(r_{M|X},\;r_{Y|X}\right),$$

which is capable of capturing the presence of mediation in a hypothesis testing framework. Boca et al. [14] evaluate the significance of this test statistic using permutation testing.

### 2.3. Motivation for Using Energy Statistics, dCor and pdCor

Székely and Rizzo introduced a series of non-parametric tests of covariance and correlation based on energy statistics, the theoretical understanding that observations are governed by a statistical potential “energy” which is zero if and only if the underlying statistical null hypothesis is true [18]. In this context, assessments and relationships of objects are made by first calculating corresponding distances of objects and all hypothesis testing and inferences are made based on these initial distances. This allows us to compare objects against each other using their relative distance and without any knowledge about their size or other properties. In this publication, we make use of distance correlation, dCor [19], and partial distance correlation, pdCor [20], which are available in R package energy [21].

The dCor test of multivariate independence, based on the corresponding sample distance covariance dCov, has unique properties of measuring dependence. The definitions of these parallel those of the classical Pearson product moment correlation *ρ* with the major difference being that the centered product moment transformation is applied to the distance matrices rather than data vectors. This test of independence can be easily applied in arbitrary dimensions—not necessarily equal—and without assumptions such as normality in the product–moment correlation counterpart. The dCov and dCor have been shown to be more powerful than the parametric counterparts, especially for nonlinear dependence structures. The practicality of applying a test and measure of dependence in high dimensions that is not only easy to apply, but also easy and intuitive to interpret, is invaluable.

The Pearson partial correlation which measures the partial correlation in vectors *x* and *y*, controlling for *z*, is described with the following partial correlation coefficient:

(5) $$r\left(x,y;z\right)=\frac{r\left(x,y\right)-r\left(x,z\right)r\left(y,z\right)}{\sqrt{1-r{\left(x,z\right)}^{2}}\sqrt{1-r{\left(x,y\right)}^{2}}},$$

where $r\left(x,y\right)$ is the Pearson sample correlation and x, y, and z are one-dimensional data vectors. As an extension of the Pearson partial correlation and, in much the same way, Székely and Rizzo [20] introduced partial distance correlation pdCor:

(6) $$pdCor\left(X,Y;Z\right)=\frac{R^{*}\left(X,Y\right)-R^{*}\left(X,Z\right)R^{*}\left(Y,Z\right)}{\sqrt{1-R^{*}{\left(X,Z\right)}^{2}}\sqrt{1-R^{*}{\left(X,Y\right)}^{2}}},$$

where $R^{*}\left(X,Y\right),\;R^{*}\left(X,Z\right),\;and\;R^{*}\left(Y,Z\right)$ denote the bias corrected distance correlation. We suggest a review of Szeékely and Rizzo, 2007 [22], 2013 [23], and 2014 [20] for a theoretical basis and deeper understanding of the methods.

### 2.4. Multivariate Omnibus Distance Mediation Analysis Statistic

In modeling relationships between multivariate variables, we must be able to express the relationship between those in terms similar to the Pearson correlations and partial correlations. To do so, we use distance correlation and partial distance correlation statistics that are capable of capturing relationships between vector-valued random variables. These statistics naturally flow from the definition of Pearson correlation by allowing a distance metric (such as Euclidean distance, or specialized distances for microbiome data) to serve as a sufficient statistic for the dependence relationship within each random vector. Using these, the multivariate omnibus distance mediation analysis (MODIMA) test statistic is as follows:

(7) $$S_{d}\left(d_{X}\left(X\right),d_{M}\left(M\right),d_{Y}\left(Y\right)\right)=dCor\left(d_{X}\left(X\right),\;d_{M}\left(M\right)\right)pdCor\left(d_{X}\left(Y\right),d_{M}\left(M\right)|d_{Y}\left(X\right)\right),$$

where $d\left(.\right)$ are appropriate pairwise distance matrices computed from the potentially multivariate observations of exposure, X, mediator, M, and response, Y.

**Figure 1:**
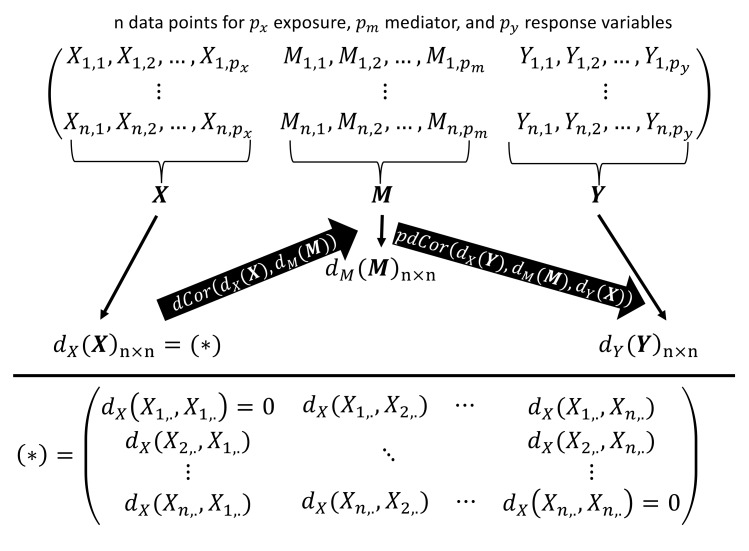
Visual description of the multivariate omnibus distance mediation analysis (MODIMA) test statistic. First, the n×n distance matrices are calculated from just the exposure, $d_{X}\left(X\right)$, just the mediator, $d_{M}\left(M\right)$, and just the response, $d_{Y}\left(Y\right)$, variables. Using these pairwise matrices, distance correlation (dCor) of the exposure–mediator and partial distance correlation (pdCor) of mediator–response are calculated using R package energy [21]. Product of dCor and pdCor results in MODIMA test statistic.

For a more intuitive understanding of the MODIMA method, consider the illustration in Figure 1. Suppose our data consists of n observations for $p_{x}$ exposure, $p_{m}$ mediator, and $p_{y}$ response variables. The test statistic is obtained by first calculating the n×n distance matrices from just the exposure, $d_{X}\left(X\right)$, just the mediator, $d_{M}\left(M\right)$, and just the response, $d_{Y}\left(Y\right)$, variables. Note that the distance (or dissimilarity) metric can potentially be different for each of these, as appropriate given the nature of these variables. The distance matrices are then used to compute the distance correlation between the exposure and mediator and the partial correlation between the mediator and the response, given the exposure. These two quantities are then multiplied together to obtain the test statistic in Equation (7).

### 2.5. MODIMA Permutation Testing

The permutation testing approach for the MODIMA method follows that of Boca et al. [14]. In short, to obtain the empirical distribution of the MODIMA test statistic $S_{d}$ under the null hypothesis, either the relationship between the exposure and mediator, or the conditional relationship between the response and the mediator has to be scrambled. Thus, if the magnitude of the first is smaller than that of the second, we permute the rows and columns of the $d_{X}\left(X\right)$ matrix and re-compute the test statistic $S_{d}\left(i\right)$. Conversely, a permutation of the response distance matrix $d_{X}\left(Y\right)$ is performed to re-compute the test statistic if its partial correlation with the mediator is greater. The *p*-value of the observed $S_{d}$ is obtained as the frequency with which the permuted statistic exceeds the observed in q permutations, P=1q∑i=1q1(Sd≤Sdi). Permutation testing is generally a powerful way to simulate from the null distribution; however, it is often hard to compute estimates of extremely small *p*-values. Although not implemented in this version of MODIMA, solutions exist to estimate small *p*-values based on fitting extreme value distributions to the permuted test statistics (e.g., application of Pareto distribution to permutation testing [24]). Reference R language implementation of the MODIMA test is available at https://github.com/Alekseyenko/MODIMA.

### 2.6. Empirical Evaluation Simulation

**Figure 2:**
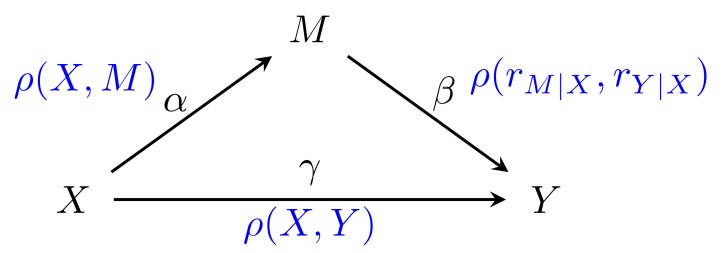
Linear model parameterization of the single mediator model showing exposure, X, mediator, M, and response, Y, and the appropriate correlation and conditional correlation coefficients.

Single mediator. To assess statistical properties of the proposed omnibus method and compare it to existing methods, we simulated data where exposure, X, mediator, M, and response, Y, were normally distributed and followed the linear model formalism of the single mediator model (Figure 2). In this case, we varied the parameters α, β, and γ as follows: $\alpha =\left\{0,\;0.25,\;0.5,\;0.75,\;1\right\}$, $\beta =\left\{0,\;0.25,\;0.5,\;0.75,\;1\right\}$, and $\gamma =\left\{0,\;0.1,\;0.25,\;0.5\right\}$. Under each combination of parameters, we simulated datasets with a varying number of observations, $n=\left\{20,\;50,\;100,\;150,\;200\right\}$. To ensure unit variance, the standard deviations of X, M, and Y  were fixed at $SD_{X,\;M,\;Y}=1$. Euclidean distance was used to compute the distance matrices. For each combination of parameters, we generated 1000 datasets for a total of 250,000 datasets. Each dataset was analyzed using our reference implementation of MODIMA as well as previous methods proposed by Sampson [14] and Chen [17].

Multiple mediators. In the presence of multiple mediators, we simulated data where exposure, X, and response, Y, were normally distributed and parameters α, β,andγ were set just as described in the single mediator case. Here, the standard deviation of X, $SD_{X}=1$ and that of Y, $SD_{Y}=0.01$ and Euclidean distances were computed on each. M was generated using a mixture of two datasets (saliva and tonsils) from the National Institutes of Health (NIH) Human Microbiome Project [25] sourced through R package HMP [26]. Saliva and tonsil datasets contain abundance data consisting of 21 taxa on 24 subjects. A proportion of used data from each dataset was determined as a function of α parameter and sample size. The abundance data were used to compute the parameter of the Dirichlet-multinomial distribution [27] modeling over-dispersion and used to generate random Dirichlet-multinomial samples for each iteration of the simulation. Additionally, rooted trees with 21 tips were generated using R package ape [28] and used for the computation of the tree-based weighted UniFrac [29] distance of the mediator. Non-tree-based distance methods of Jensen–Shannon divergence (JSD) [30] and Bray were computed for the mediators using R package phyloseq [31] and vegan [32], respectively. Parameters α, β, and γ were set in a similar fashion to the single mediator simulation and sample sizes were set to $n=\left\{20,\;50,\;100,\;150\right\}$.

Details of the simulation are available as a knitted R Markdown file at https://github.com/Alekseyenko/MODIMA.

## 3. Results

### 3.1. Empirical Evaluation of MODIMA

**Figure 3:**
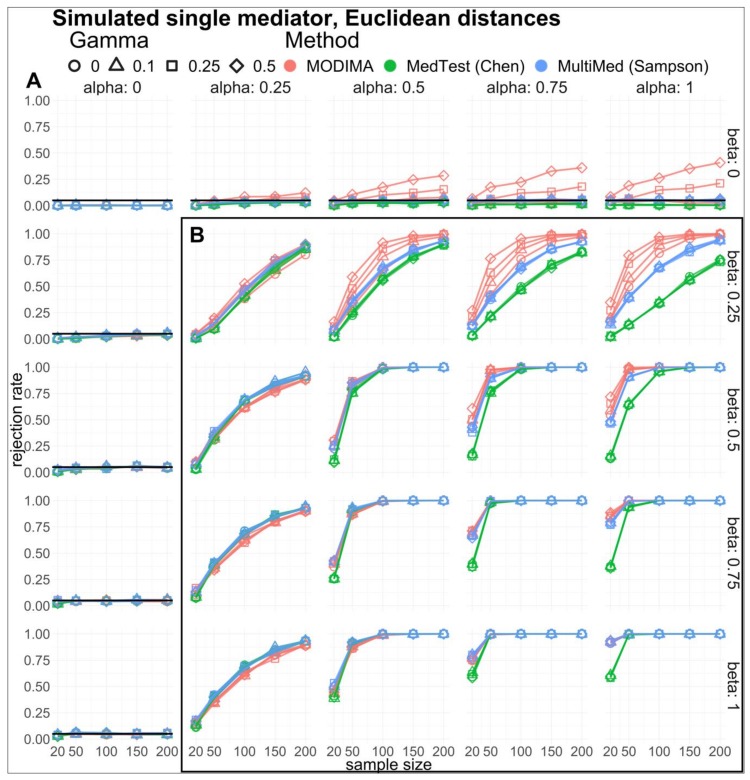
Simulated single mediator results. (**A**) Type I error and (**B**) power are estimated by simulation at varying α,β,γ, and sample size. Comparison between the approach proposed here and other methods proposed by Sampson (MultiMed) [14] and Chen (MedTest) [17] is portrayed using red, blue, and green, respectively. Point shapes portray the various degrees of X−Y relationship, γ. Horizontal black lines in (**A**) represent 0.05 type I error rate commonly used.

**Figure 4:**
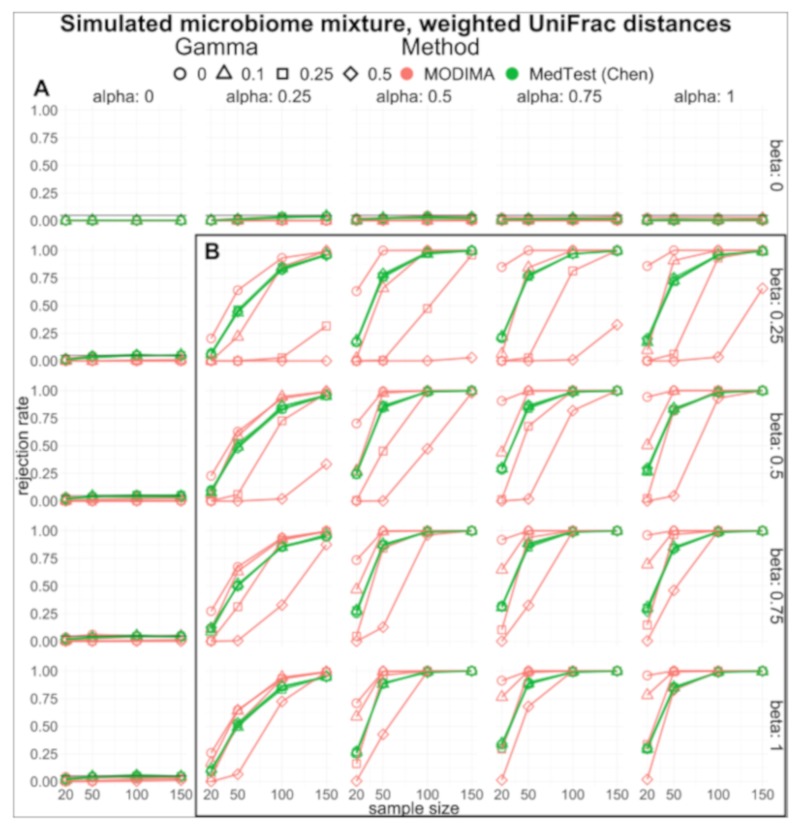
Simulated microbiome mixture results. (**A**) Type I error and (**B**) power are estimated by simulation at varying α,β,γ, and sample size. Shown here are the weighted UniFrac [29] distance metrics computed on microbiome matrices. Comparison between the approach proposed here and Chen (MedTest) [17] is portrayed using red and green, respectively. Point shapes portray the various degrees of X−Y relationship, γ. Horizontal black lines in (**A**) represent 0.05 type I error rate commonly used. Simulated microbiome mixture results using other distance measures are provided in Figures S1 and S2, Supplementary Materials.

The simulation results comprise the sample size-dependent type I error rates and power as a fraction of rejected null hypotheses at a significance threshold of 0.05 for each test (Figure 3 and Figure 4). In the case of the single mediator, when the null hypothesis is true (Figure 3A), the association of the exposure with the mediator (α=0) or the effect of the mediator on the response (β=0) are absent. A test properly controlling type I error rate is expected to have a fraction of rejections equal to the nominal error rate (0.05, in this case). In the cases of β=0, as α and γ are increased, we observe inflation of this type I error rate for MODIMA; however, Sampson [14] and Chen [17] methods often display overly conservative type I error rates. This effect has been previously described [20] and is related to the fact that zero partial distance correlation does not correspond to conditional independence. We review this point further in the Discussion section. Our proposed method is able to demonstrate equal or better power (Figure 3B), often increasing power with the increase of mediating effect. Within a few selected parameters, all three methods performed equally. Notably, the MedTest method often shows the least power and does not perform well when the association of the mediator with the exposure is much higher than the association of the mediator with the response (Figure 3B, α=1 column). In fact, that appears to be the most challenging condition for all approaches to make the necessary rejections. Our approach maintains the best performance in that instance.

In the case of the many mediators, we generated mixtures of microbiome data to be used as mediators. We computed distance metrics of Bray, Jensen–Shannon divergence (JSD), and UniFrac and present the latter here in Figure 4. The former two can be found as part of our supplementary data. When the null hypothesis is true (Figure 4A), both MODIMA and MedTest are able to maintain rejection rates. The behavior of MODIMA to inflate the rejection rates under β=0 observed in the single mediator case is no longer present in the multiple mediator simulation case. Power curves (Figure 4B) show MODIMA excelling under certain scenarios, whereas MedTest displays better power in others. Overall, MODIMA excels under scenarios where the X**-**Y relationship noted by γ is smaller or, in other words, relatively small to no direct relationship between the exposure and outcome. Under the smaller sample sizes, we often see MODIMA performing slightly better.

We next demonstrate the application of MODIMA in two empirical examples.

### 3.2. Application Example 1: Microbiome-Mediated Responses to Subtherapeutic Antibiotic Treatment Influencing Body Fat

Antibiotics have undoubtedly provided remarkable public health benefits in the last century. During that same time span, we see a marked increase in antibiotics use across many populations [33]. Furthermore, we see the largest use of antibacterial agents within the animal farming industry, often exclusively used in low doses to stimulate weight gain in farm animals [34]. There is growing concern about the effects from the long-term use of antibiotics and antibacterial agents [35,36]. Here, we build on the evidence on phenotypic and microbial responses to early-life subtherapeutic antibiotic treatment using murine models expanding on findings presented by Cho et al. [37].

In each experiment, each study group (control or antibiotic(s)) was composed of ten mice. The mice were allowed ad libitum access to food and water and fed standard laboratory chow. Beginning on day 28 of life, mice were given water or water containing one of the following antibiotic regimens: penicillin VK, vancomycin, penicillin VK plus vancomycin, and chlortetracycline, each at doses equivalent to 1 μg antibiotic per g body weight. On a weekly basis, mice were weighed three times, food intake measured, and fecal pellets collected. Dual energy X-ray absorptiometry (DEXA) and a 7 Tesla MRI system were used to collect animal fat composition, lean body mass, percent body fat, and bone mineral density. The IDEAL Dixon method based on chemical shift properties was used to separate MRI images into fat and lean tissue [38]. Weight values were calculated from MRI-determined fat percent to validate scale weight. The microbiome composition was established by sequencing of the v3 region of the 16S rRNA gene using 454-FLX Titanium chemistry (Roche, Bradford, CT, USA). Preprocessing was performed using QIIME pipeline [39] at a 97% similarity threshold.

A total of 96 samples (50 cecal and 46 fecal) across 50 animals were used for all downstream analysis, resulting in 6547 unique taxa. Four antibiotic regimens of penicillin *n* = 10, vancomycin *n* = 10, penicillin plus vancomycin *n* = 10, and chlortetracycline *n* = 10 were used for comparison with controls *n* = 10. For each subject, cecal and fecal microbiome data were available.

Jensen–Shannon divergence (JSD) distances were computed for microbiota (mediator) and Euclidean distances for antibiotic use (exposure) as well as percent fat (outcome). The mediating relationship of the combined cecal and fecal microbial composition between antibiotic intake and percent fat resulted in a MODIMA statistic of 0.002 (*p* = 0.99). Assessing the relationship of antibiotic use and percent fat, we see a bias-corrected distance correlation (bcdCor) estimate of 0.173. Likewise, we see dCor estimates of microbiota (cecal and fecal combined) to antibiotic use and percent fat to be 0.113 and 0.000. Partial distance correlation (pdCor) between the relationship of antibiotic use, percent fat, and microbiota (cecal and fecal JSD) is calculated to be 0.021 (*p* = 0.46).

**Figure 5:**
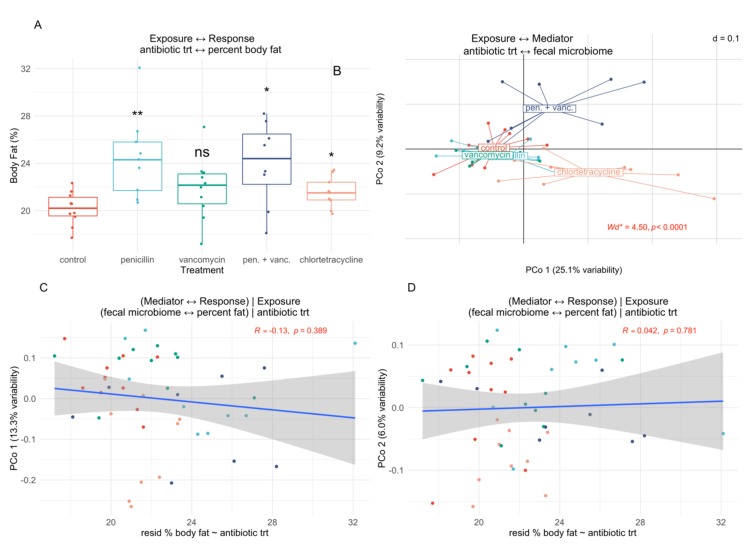
Empirical example 1, fecal microbiome-mediated responses to antibiotic treatment. (**A**) shows association of antibiotic treatment and percent bodyfat; (**B**) shows percent body fat was significantly increased in all antibiotic groups with the exception of vancomycin; *p*-values are noted by symbols **, *, and ns corresponding to *p* < 0.01, < 0.05, and not significant, respectively; association of antibiotic treatment with microbiota PCo axes 1 and 2, as well as Welch statistic and *p*-value suitable for analysis of microbiome data, $W_{d}^{*}$ [40]; (**C**) and (**D**) show, respectively, the lack of association between microbiota and body fat using PCo axes 1 and 2, removing any effect of antibiotic treatment.

Fecal and cecal samples were also analyzed separately. We observe MODIMA statistics of 0.008 (*p* = 0.81) and 0.007 (*p* = 0.72) for fecal (Figure 5) and cecal (Figure S3, Supplementary Materials) samples, respectively. Using the provided estimates, we see that variable pairs do show mild distance correlation for both fecal and cecal (Additional Files 1 at https://github.com/alekseyenko/MODIMA). Partial distance correlation computations remain negligible, 0.026 (*p* = 0.57) and 0.018 (*p* = 0.59) for fecal and cecal samples, respectively, and these total effects can be seen in Figure 5C,D and Figure S3C,D (Supplementary Materials at https://github.com/alekseyenko/MODIMA)).

Further antibiotic-specific comparisons are made between individual antibiotic therapies and control. Most notable changes are seen when cecal and fecal are assessed individually with a specific antibiotic treatment. An assessment of cecal and fecal microbiome mediation of the penicillin versus control exposure results in MODIMA statistics of 0.019 (*p* = 0.28) and 0.034 (*p* = 0.09), respectively. Partial distance correlations are observed to be 0.023 and 0.110 for cecal and fecal. For fecal specifically, although mild correlation is present, and when assessed for mediation using MODIMA, effects are not detectable. Comparison of chlortetracycline and control using samples from fecal microbiome revealed the largest and only statistically significant mediation, with MODIMA statistic of 0.141 (*p* = 0.016).

Data and analysis for this application are available at https://github.com/Alekseyenko/MODIMA.

### 3.3. Application Example 2: Microbiome-Mediated Responses to Dietary Fiber Intake Influencing Body Mass Index (BMI)

A growing body of evidence suggests that diet influences the compositional diversity of gut bacteria [8]. We also see an association between changes in gut bacteria diversity and human health, such as obesity [41]. Through a study of diet and 16S ribosomal DNA (rDNA) fecal samples, Wu et al. (2011) reported that long-term diet was strongly associated with enterotype clustering [42]; here, we briefly describe their methods. Healthy human subjects (*n* = 98) were enrolled in a cross-sectional study where long-term diet information was collected using self-reported questionnaires assessing usual dietary composition over the preceding year. Diet information was subsequently converted to a list of 214 nutrient categories and their corresponding intake amounts. Stool samples were collected, frozen immediately (−80 °C), processed using MoBio PowerSoil kits, amplified V1–V2 region primers targeting bacterial 16S genes, and sequenced using 454/Roche. Sequences were denoised using QIIME pipeline [39] following default settings. Other demographic information including body mass index (BMI) was collected upon enrollment. Weighted and unweighted UniFrac distances for microbial communities were calculated and used for downstream analyses.

**Figure 6:**
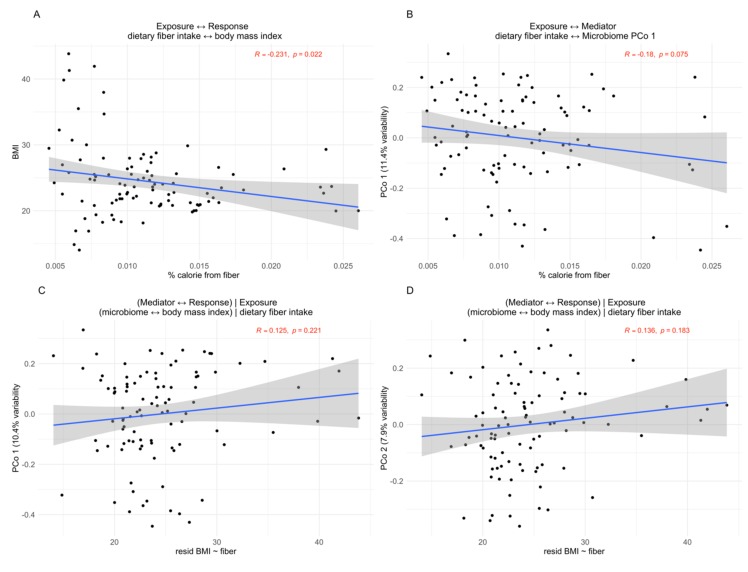
Empirical example 2, microbiome-mediated responses of body mass index (BMI) to dietary fiber intake. (**A**) shows association of fiber intake with outcome of BMI; (**B**) shows association of fiber intake with microbiota PCo axis 1, PCo axis 2 results can be seen in Figure S4, Supplementary Materials; (**C** and **D**) demonstrate, respectively, the lack of association between microbiota and BMI using PCo axes 1 and 2, removing any effect of dietary fiber intake. Pearson correlation coefficients and *p*-values are shown in red.

Wu et al. [42] previously reported a strong inverse association between body fat intake and microbial taxa (Spearman ρ=−0.68, p<0.0001).  These same microbial taxa were observed to be associated with BMI (PERMANOVA, unweighted *p* = 0.001, weighted *p* = 0.145). Here, we assessed the influence of dietary fiber intake on BMI mediated by microbiota. The correlation of percent fiber intake (exposure) and BMI (response) is small yet significant (Figure 6A), and fiber intake and microbiota (mediator) is small and approaching statistical significance (Figure 6B). Mediator–response relationships (total effects) are modeled in Figure 6C,D using first and second principal coordinates and residuals of exposure–response. Zhang et al. [17] applied their omnibus mediation method MedTest using multiple distance metrics to assess this mediation and showed a permutation-based *p*-value of 0.0309. Furthermore, mediation by individual taxa at different ranks was assessed. Zhang et al. [17] observed three ranks to be significant: family Lachnospiraceae (*p* = 0.0129), genus *Lachnospira* of the family Lachnospiraceae (*p* = 0.0430), and family Ruminococcaceae (*p* = 0.0468). We applied our proposed distance mediation analysis methods to this dataset and present our findings.

Distance metrics of Jensen–Shannon divergence (JSD), Bray–Curtis, Jaccard, and UniFrac (unweighted, weighted, and generalized) were computed using distance function in R package phyloseq (version 1.26.1) [31], vegdist function in R package vegan (version 2.5.4) [32], and GUniFrac function in R package GUniFrac (version 1.1) [43], respectively, following similar methodologies as presented by Zhang et al. [17]. Distance correlation (dCor) estimates between pairs of exposure, mediator, and response showed no evidence of correlation using any of the distance metrics (data not presented here but are available as Additional Files 2 at https://github.com/Alekseyenko/MODIMA). In a likewise fashion, the partial distance correlation estimate between fiber intake, microbiome, and BMI was not indicative of any correlation (estimate using JSD applied to mediator = 0.029, *p* = 0.04). MODIMA was applied using various distance metrics. Table 1 summarizes the results for MODIMA *p*-values for each distance metric as well as Bonferroni-adjusted test. As shown, we see that only the Jaccard metric shows a significant *p*-value at α of 0.05, with Bray–Curtis and unweighted UniFrac distances approaching the significance threshold. We further observe MODIMA resulting in lower *p*-values than MedTest in single-distance mediation tests with the exception of Jaccard.

To further assess potentially mediating taxa within this dataset, we sliced the released phylogenetic rooted tree using library phytools (version 0.6.60) [44], beginning at root (slice 0.01) to the height of 1 at 0.01 increments. Each of these slices resulted in subtree clades that were used for comparison testing. Each arbitrary slice resulted in clades that held taxa unique to them. We saw that, aside from a handful of the clades within certain slices, no mediation was observed with MedTest. This suggests that, if present, the mediation effect of the microbiota on the fiber intake and BMI relationship is likely small enough to be undetectable within the given sample size.

Data and analysis for this application are available at https://github.com/Alekseyenko/MODIMA.

## 4. Discussion

In this article, we developed a framework for multivariate omnibus distance mediation analysis (MODIMA). Although the proposed methods have wide applications to various data types, we specifically showed their robustness in high-dimensional settings by applying them to novel and previously published microbiome data. In simulations, we showed that our method to detect mediation under various scenarios is more powerful than previously published work. Simulations showed that MODIMA holds empirical type I error rates at the desired nominal significance level under the multiple mediator case.

Clearly, any analysis based on distances should not blindly and thoughtlessly pick the metric to be used. Ideally, the structure of the data and the analyst’s intuition about the problem should guide the selection of an appropriate measure. A universally best distance measure may not exist for all problems and different data may result in different best performing distances, in terms of sensitivity and specificity. For example, although very popular and used in many studies, weighted UniFrac distance failed to result in a rejection in application example 2. This does not imply that this distance is universally bad. Its performance in combination with that of unweighted UniFrac is possibly telling us that beyond the phylogenetic signal the relative abundances are not informative of the mediation that may or may not exist in the data. A limitation of our method relative to MedTest is that MODIMA works on a specific distance metric rather than pooling analyses from multiple metrics. A future improvement to the method should incorporate the ability to use multiple distances. In the interim, a test-and-adjust approach can be used with MODIMA with multiple distances.

With regard to empirical power as the exposure–mediator and mediator–outcome effects are increased, MODIMA displayed increasing empirical power characteristics relative to other methods. We further see that there was increased power as sample size increased from 20 to 200, a typical expectation. It should be noted that most ‘omics investigations operate on the lower end of that sample size spectrum; therefore, the ability to correctly detect differences under small sample scenarios is important.

In both of our empirical examples, although the mediating effect of the microbiota was plausible, we failed to detect the mediation. This is unsurprising for datasets of such small size and relatively small total effect of the exposure on the response (e.g., dCor = 14–17% for antibiotics–percent body fat and dCor = 9% for fiber–BMI). The microbiome in dietary fiber effect on the body mass index example demonstrates a discrepancy observed between our method and an alternative approach. Through additional analyses, we demonstrate that either the effect is again too small to be detected in a dataset of this size, or that it may be absent altogether.

Our application of the distance correlation and partial distance correlation metrics to the problem of modeling distance mediation illustrates somewhat unintuitive notions relating dependence and correlation in the context of causal analyses. First, the absence of partial correlation does not automatically imply the absence of partial dependence. The equivalence of partial correlation and conditional dependence is only true for a multivariate normal family of distributions. Furthermore, distance correlation methods demonstrate non-zero partial correlation (even asymptotically) in certain scenarios with conditionally independent univariate normal variables [20]. Specifically, as is relevant to the mediation analysis, consider X is a standard normal random variable and M and Y are each an independent linear combination of X and another standard normal variate. In that case, pdCor(M, Y | X) > 0, indicating the presence of partial distance correlation. This suggests that the notion of conditional dependence captured by partial distance correlation is different from that intuitively expected. Székely and Rizzo [20] suggest that partial distance correlation implies that there exists a pair of U-equivalent random variables that are in fact conditionally independent. The extent to which a lack of correspondence between conditional independence and zero partial distance correlation is a problem with multivariate data is unknown at the moment. However, it is easy to see via simulation that adding additional mediators uncorrelated to the exposure will decrease the population values of pdCor, which in finite sample size results in fewer rejections and thus less inflated type I errors. This is demonstrated in our multiple mediator simulations, where adding a moderate number of mediators results in better type I error control in simulation under the null hypothesis (Figure 4A).

Another potential pitfall of multivariate mediation analysis pertains to the interpretations of significant mediation results. Consider, for example, a scenario where X is a true cause for both Y and M1, while independent of M2, which is a true cause of Y. Although no univariate mediation relationships exist under this scenario, multivariately M = (M1, M2) is not conditionally independent of Y, given X and multivariate (distance) mediation does exist. This suggests that, whenever multivariate distance mediation is established, further interpretations of this relationship must be treated with caution in order not to attribute this relationship to any individual univariate marginals of the X, M, Y triple, but to treat this relationship as existing in the joint distribution.

Omnibus mediation analysis with ’omics-sized mediators is the first step towards enabling top-down approaches in genomic data. As opposed to the more widely used methods that integrate the univariate signals of individual measurements of microbes, gene expression, or genetic variants, the top-down approach starts with the collective effect of those and prunes the individual measurements down to a small set of most important ones. The significance of this approach is that top-down thinking allows for capturing effects, such as epistasis and otherwise complexly intertwined relationships. We envision that future versions of the omnibus mediation approach of this paper and of alternative approaches will allow to assign importance to components in addition to assessing the overall effect of the entire collection.

## Figures and Tables

**Table 1:** MODIMA and MedTest *p*-values for various distance metrics.

	Jensen–Shannon	Bray–Curtis	Jaccard	UniFrac	WUniFrac	GUniFrac	Bonferroni
MODIMA	0.1074	0.0974	0.0321	0.0706	0.4645	0.2543	0.1926
*p*-value							
MedTest [17]	0.5423	0.5568	0.0082	0.0901	0.7859	0.5768	0.0492
*p*-value							

## Supplementary Materials

Supplementary materials include source code for methods, simulation studies, and application examples and are freely available at https://github.com/Alekseyenko/MODIMA.
